# Chip-Based Digital PCR Approach Provides A Sensitive and Cost-Effective Single-Day Screening Tool for Common Fetal Aneuploidies—A Proof of Concept Study

**DOI:** 10.3390/ijms20215486

**Published:** 2019-11-04

**Authors:** Anna Nykel, Marcin Kaszkowiak, Wojciech Fendler, Agnieszka Gach

**Affiliations:** 1 Department of Genetics, Polish Mother’s Memorial Hospital Research Institute, 93-338 Lodz, Poland; ania.nykel@gmail.com; 2 Department of Biostatistics and Translational Medicine, Medical University of Lodz, 92-215 Lodz, Poland; mgkaszkowiak@gmail.com (M.K.); fendler.wojciech@gmail.com (W.F.); 3 Department of Radiation Oncology, Dana-Farber Cancer-Institute, Boston, MA 02215-5450, USA

**Keywords:** digital PCR, rapid aneuploidy screening, prenatal diagnosis

## Abstract

In the prenatal period, the copy number aberrations of chromosomes 13, 18, 21, X and Y account for over 80% of the clinically significant chromosome abnormalities. Classical cytogenetic analysis is the gold standard in invasive prenatal diagnostics but the long test waiting time affects its clinical utility. Several molecular rapid tests have been developed and employed in clinical practice, however all have substantial drawbacks. The aim of the study was to design and evaluate an optimized tool for rapid molecular detection of fetal aneuploidies. We established a novel single-day method using a chip-based platform, the QuantStudio 3D Digital PCR system. In order to assess the clinical usefulness of our screening test, we analyzed 133 prenatal samples. The difference in distributions of euploid and aneuploid samples identified the ploidy of each of the target chromosomes with high precision. The distribution of the chromosome ratio for euploid and aneuploid samples showed a statistically significant result (*p* = 0.003 for trisomy 13, *p* = 0.001 for trisomies 18 and 21, Mann–Whitney *U* test). Our results suggest that this novel chip-based approach provides a tool for rapid, technically simple, cost-effective screening for common fetal aneuploidies.

## 1. Introduction

Approximately 3 to 5% of all clinically recognized pregnancies are complicated by birth defects or genetic disorders [1]. Maternal age, fetal nuchal translucency (NT) thickness and maternal serum free β-human chorionic gonadotropin (β-hCG) and pregnancy-associated plasma protein-A (PAPP-A) are the key indicators for aneuploidy risk calculation at 11 to 13 + 6 weeks of gestation [2]. For high risk pregnancies diagnostic prenatal testing can be performed by invasive sampling and karyotyping [3]. The incidence of aneuploidies in clinically diagnosed early miscarriages is greater than 50%. It is estimated that fetal aneuploidies account for 6–11% of all stillbirths and neonatal deaths [2]. 13, 18, 21, X, Y chromosome aneuploidies are the most common aberrations and account for more than 80% of the clinically significant prenatally diagnosed chromosomal abnormalities [4]. The incidence of trisomy depends on maternal age and shows geographical differences. The most common trisomy is Down syndrome, or trisomy 21, its prevalence in Europe is estimated at 20.15 (19.85–20.46). Trisomy 13 and 18 have a much lower incidence, 1.77 (1.68–1.86) and 4.67 (4.52–4.82) respectively [5].

Classical cytogenetic analysis is the gold standard in invasive prenatal diagnosis. The main disadvantage of this method is the long test waiting time for the result, high workload and high cost. Thus, several rapid tests have been developed and employed in clinical practice, among others: fluorescent in situ hybridization (FISH), quantitative fluorescence PCR (QF-PCR) and multiplex ligation-dependent probe amplification (MLPA). They allow aneuploidy screening within 1 or 2 days [6]. However, all three methods have substantial drawbacks. FISH is a labor-intensive technique, relatively costly. In addition, it requires technical expertise and overnight hybridization step, which extends the procedure [7,8]. Compared to FISH, PCR-based methods are more suited to a high throughput diagnostic. QF-PCR may have higher sensitivity compared with the other methods. However, the main limitations are the necessity to use standardized references, complex calculations, dependence on the population genetics (amplification of polymorphic microsatellite markers) and the procedure sensitivity even to minor procedure changes [9]. The MPLA method overcomes the potential problem with amplification of polymorphic microsatellite markers, yet overnight hybridization step precludes a single-day mode. In addition, both QF-PCR and MLPA methods require a post-PCR step for quantitative analysis of the products using an expensive genetic analyzer [8].

Digital PCR (dPCR) is a relatively new technique. The concept of digital PCR was first described in the late 1990s [10]. Since then, digital PCR has been used for DNA quantification for a wide range of applications in genetic testing. Digital PCR method is based on three main points: the partition of the target, PCR on each single molecule in thousands of simultaneous reactions and Poisson statistics. Data analysis is based on the calculation of the absolute concentration of target DNA in copies/μL of reaction from the number of positive and negative partitions. Based on Poisson statistics copy number of target per microliter (*T*) is calculated with the following equation, *P* refers to the number of PCR positive partitions, *N* is the total number of partitions, *Vp* is the volume of partition and *D* is the dilution factor during DNA sample adjustment and preparation of the reaction mixture [11,12].

$$T=\frac{-D}{V_{p}}\times \;\ln\left(1-\frac{P}{N}\right)$$

The random nature of the distribution of molecules in the partitions makes the measurement accuracy both predictable and precise compared to real-time PCR [9]. The most obvious advantage of digital PCR technology is the possibility to obtain accurate absolute target concentration without standards or routine calibration. Moreover, the decreased dependence on PCR efficiency makes dPCR extremely suitable technology for low quality samples used in prenatal testing.

There is a multitude of digital PCR platforms currently in use. The one possible approach uses microfluidic chambers to split the samples, the second is based on emulsion PCR to clonally amplify templates in the presence of beads and the third method is based on water-in-oil droplets [13,14,15,16]. However, these technologies have relatively high equipment and exploitation costs. The introduction of the chip-based QuantStudio 3D Digital PCR system featuring low equipment and operating costs, compatible with TaqMan^®^ assays and relative ease of use, makes it possible to popularize dPCR technology in medium-throughput laboratories [11].

The aim of this study was to assess the clinical utility of chip-based dPCR system in identification of the most common aneuploidies in rapid screening.

## 2. Results

### 2.1. Workflow of the Chip-Based Digital PCR Experiment

**Figure 1:**
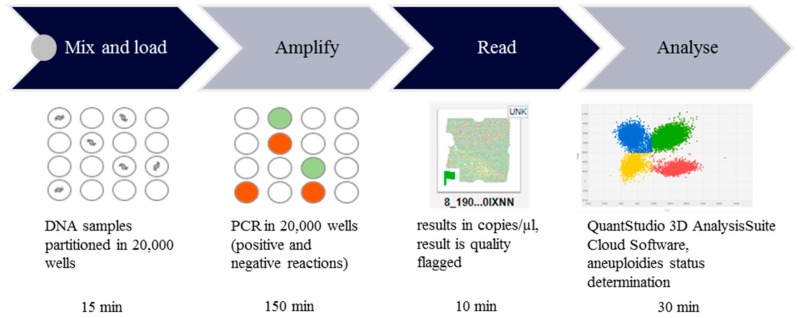
Workflow of the chip-based digital PCR experiment. Workflow of chip-based digital PCR (dPCR) is based on four stages: PCR reaction mix preparation, seal and load chips, PCR amplification and fluorescence detection. Data analysis with quality control was performed and aneuploidies status was determined. Results are obtained within 4 h. The small gray circle corresponds to the pipetting step at the beginning of the procedure. The circle with wavy line represents the DNA partition in the wells on the chip. The green and orange circle indicate the positive and negative PCR reactions. Three colors in the plot corresponds to the FAM signal (blue), VIC signal (red), both FAM and VIC signal (green) and no signal (yellow).

We established and validated a novel chip-based approach to reliably distinguish between euploid and aneuploid samples using TaqMan^®^ assays targeting chromosomes 13, 18, 21, X and Y in three duplex reactions. Furthermore, we performed statistical analysis to determine the optimal threshold for detecting aneuploidies. In order to assess the clinical usefulness of our screening test, we conducted analysis on 133 prenatal samples. The workflow of the chip-based digital PCR experiment is illustrated in Figure 1. Firstly, DNA was extracted from prenatal samples (amniotic fluid or chorionic villus samples (CVS)) and mixed with dPCR reagents. For copy number evaluation of chromosomes 13, 18, 21, X and Y duplex reactions with FAM and VIC labeled probes were performed. Following PCR reaction preparation, the chip is sealed and loaded. Secondly, PCR amplification in 20,000 wells on the chip is performed. Thirdly, FAM and VIC call from each well on the chip is red. Last step is based on data analysis and aneuploidies status determination through chromosomal ratio calculations.

### 2.2. Validation of the Duplex dPCR Reactions

**Figure 2:**
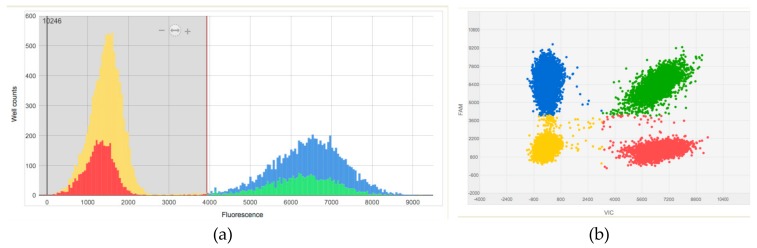
An example of a dPCR result histogram and scatterplot indicating the trisomy of chromosome 21 generated using QuantStudio 3D AnalysisSuite Cloud Software v3.1. (**a**) dPCR result histogram is generated by plotting an overview of the observed dye intensities (fluorescence) versus well count for every well on the chip. Red vertical line represents the fluorescence (call) threshold for chips calculated automatically in an Absolute Quantification project by the software. The yellow peak show unamplified wells (no target). The blue and red peak represents wells on the dPCR chip with target amplification (FAM and VIC reporter dye signal from each well). (**b**) The corresponding scatter plot showing signal from FAM reporter dye on the Y-axis against the signal from VIC reporter dye on the X-axis. All data points from every well on the dPCR chip are shown in the plot. The data points in the plot showing no signal (yellow), with only FAM reporter dye signal (blue), with only VIC reporter dye signal (red) and with both FAM and VIC reporter dye signal (green). In this study the following assays were used: probe on the chromosome 21 that generates a FAM-positive signal and probe on the reference chromosome 13, which generates a VIC-positive signal.

The application used in this study allowed us to analyze the number of copies of chromosomes 13, 18, 21, X and Y for the most common aneuploidies in the fetus. To allow large-scale use of rapid prenatal screening test, considering the costs of the study, for each patient the reaction was set in triplicate. Each of the reactions was set as a duplex reaction labeled with VIC and FAM dyes. We used a set of VIC TaqMan^®^ assays for the *MBNL2* gene on chromosome 13 and noncoding region on chromosome X. To detect chromosomes 18, 21 and Y FAM TaqMan^®^ assays for the *EHZF*, *PRDM15* and *SRY* genes respectively were performed. The obtained results indicate that regardless of the choice of probe pairs, the analysis was consistent. Therefore, we eliminated an additional probe targeting reference chromosome. We did not observe differences in results between multiplex and singleplex assays per chromosome. Finally, the duplex reactions for aneuploidies detection were set in the following pairs: probe targeting chromosome 13 (VIC) with probe targeting chromosome 21 (FAM), probe targeting chromosome 13 (VIC) with probe targeting chromosome 18 (FAM) and for analysis of sex chromosomes: duplex probe assay targeting noncoding X sequence (VIC) and *SRY* gene (FAM). The result obtained from the duplex reaction for probe targeting chromosome 21 (FAM) compared to the reference probe targeting chromosome 13 (VIC) for the trisomic sample is shown in Figure 2.

### 2.3. Determination of the Cut-Off Value for Aneuploidies

One of the important issues in dPCR data analysis is to set an appropriate threshold to determine positive and negative data results. The results of the analysis for 133 samples in this study indicate that the automatic thresholding parameter of the QuantStudio 3D was sufficient to distinguish between euploid and aneuploid samples. The data analysis and automated calling were performed using QuantStudio 3D AnalysisSuite Cloud Software. For each target chromosome, euploid and aneuploid samples ratio was calculated by dividing the number of copies/μL of the analyzed chromosome by that of the reference chromosome. The optimal threshold for distinguishing between euploid and aneuploid group was 1.15. Samples with chromosomal ratio between 0.9 and 1.1 were classified as euploid (Table 1).

### 2.4. Validation of Clinical Sample Detection Using Chip-Based dPCR

**Figure 3:**
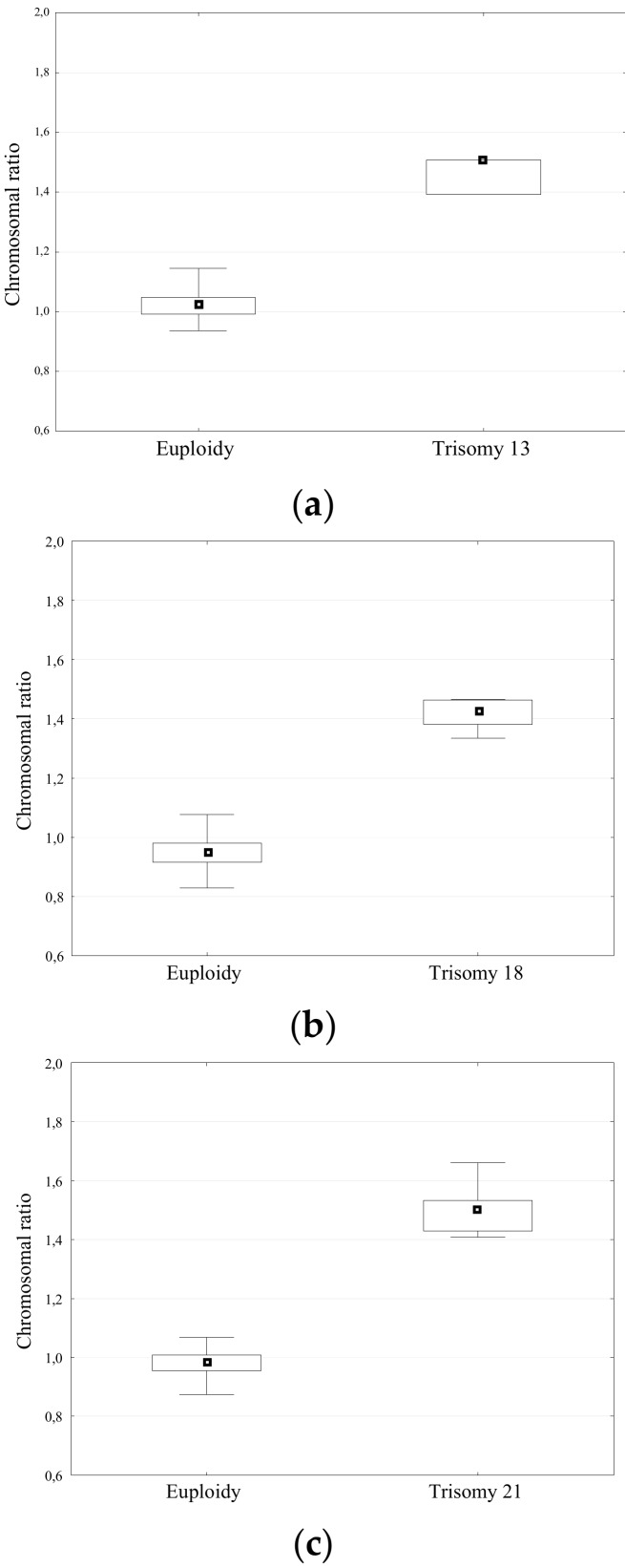
Box-and-whisker plots of the distribution of chromosome ratios in euploid and aneuploid samples. (**a**) Trisomy 13; (**b**) Trisomy 18 and (**c**) Trisomy 21. The boxes display the median (25th–75th percentiles) for the distribution; the whiskers represent the minimum and maximum value and the squares in the center of the plot represent the median.

**Figure 4:**
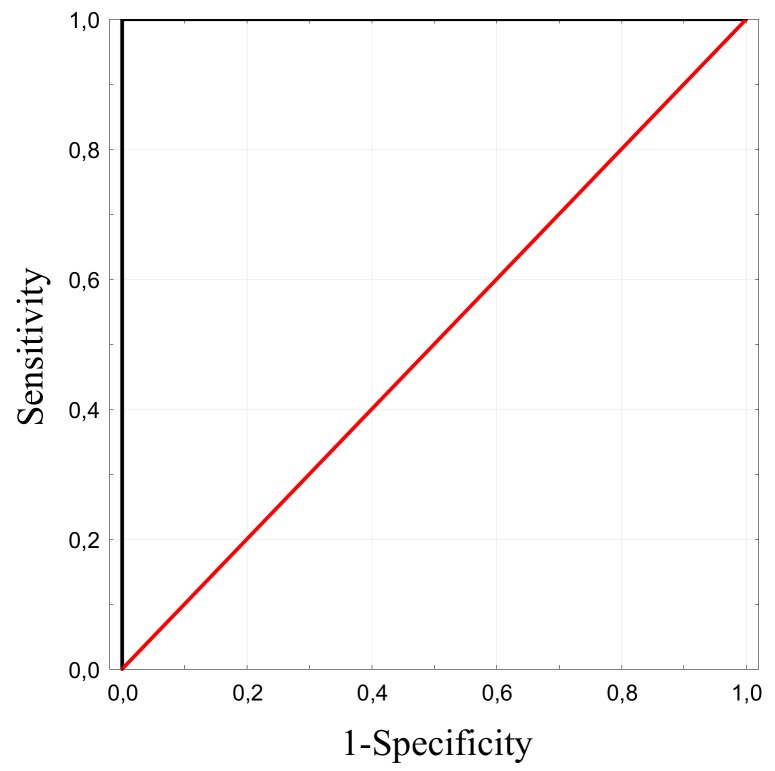
The receiver operating characteristic (ROC) curve for the chromosomal ratio as a predictor for trisomy 13, 18 and 21 (identical for each trisomy). The area under the ROC curve (AUROC) is given with its 95% confidence interval. AUROC: 1 (95%IC: 1.0; 1.0).

Of the 133 analyzed samples, chromosomal aberrations were found in 31 cases (19/106 amniotic fluid samples, 12/27 CVS). The most frequently occurring aberration was trisomy 21, identified in 17 cases. Digital PCR analysis also identified in nine cases of trisomy 18, three cases of trisomy 13 and one case of monosomy X and XXXY. Results from dPCR analysis were obtained from each sample, regardless of the quality and concentration of fetal DNA. All samples were analyzed simultaneously with two procedures: digital PCR analysis and cell culture for conventional karyotyping with GTG-banding. The results were congruent. There were no false positive or false negative results. The distribution of the chromosome ratio for euploid and aneuploid samples showed a statistically significant result (*p* = 0.003 for T13, *p* = 0.001 for T18 and T21, Mann-Whitney *U* test; Figure 3). For each trisomy screening test a receiver operating characteristic (ROC) curve of the chromosome ratio was plotted. The area under the ROC curve (AUROC: 1 (IC 95%: 1.0; 1.0)) and the shape of the curve was characteristic for an optimal screening test. The obtained specificity with optimal threshold allowed for an easy and unambiguous discrimination between euploid and aneuploid samples with 100% confidence limit (Figure 4). For each trisomy the test allowed precise separation (sensitivity and specificity were equal to 100%, AUROC = 1 (95% IC: 1.0; 1.0).

### 2.5. dPCR Sensitivity to Mosaicism and Maternal Cell Contamination

**Figure 5:**
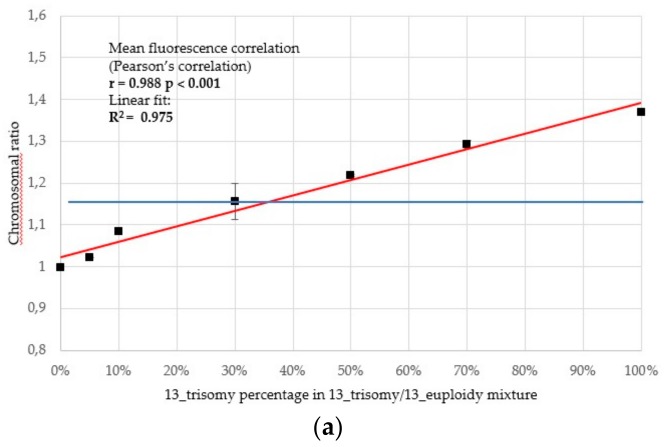

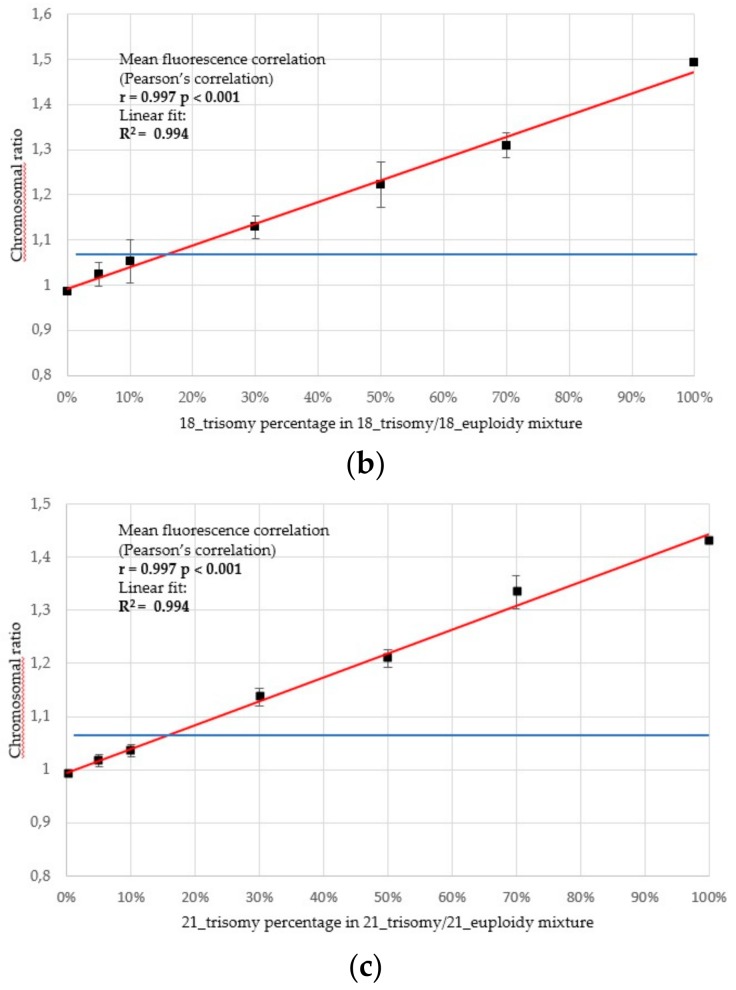
Scatter plots of serial dilutions for aneuploidies. Mixtures containing 0%, 5%, 10%, 30%, 50%, 70% and 100% of trisomies were performed. Scatter plots representing (**a**) slope ratios in 13 trisomy; (**b**) slope ratios in 18 trisomy and (**c**) slope ratios in 21 trisomy as a function of the percentage of the respective aneuploidies in the aneuploid/euploid mixture. Individual samples were analyzed in technical duplicates and averaged. The Pearson’s correlation was calculated. The red line represents an estimated linear fit (with a respective *R*^2^ value). The blue line marks a suggested cut-off point for aneuploidies detection (estimation based on Figure 3 on the basis of maximum values observed in euploid samples: 1.145 for T13, 1.068 for T21 and 1.077 for T18).

In prenatal testing mosaicism and maternal cell contamination can significantly affect the results of the analysis. To assess chip-based dPCR sensitivity and test the limits of detection, mixtures containing various proportions of samples with 13, 18 and 21 trisomy were performed (Figure 5). We tested our assays with artificial mixtures containing 5%, 10%, 30%, 50% and 70% of trisomy and analyzed the chromosomal ratios obtained for different replicates of the same point. We analyzed the correlation and linear regression between the observed and theoretical ratios for aneuploidies.

There was a strong positive correlation of expression ratios with the percentage of aneuploidy material in the serial dilution experiment. In all cases, the effect was statistically significant (Figure 5). Linear regression analysis of data shows that detection of aneuploidy is with good linear fit (*R*^2^ = 0.975 for T13, *R*^2^ = 0.994 for T18 and T21) between the measured and calculated aneuploidy content. With chip-based PCR, the overall assay variability among technical replicates (two chips per mixture) was low without normalization. A suggested cut-off point for aneuploidies detection was estimation based on the results depicted on Figure 3 on the basis of maximum values observed in euploid samples: 1.145 for T13, 1.068 for T21 and 1.077 for T18. In the dilution experiment, means with estimated 95% IC suggest that the method might identify samples as aneuploid whenever the percentage of the trisomic DNA was as low as 20% in case of trisomy 21 and 18. For trisomy 13 the detection limit was ~30%. More detailed tests to verify this observation would be however needed to evaluate performance of the assay in real-life scenarios with more confounding variables.

In our clinical practice we identified one case of karyotype-confirmed maternal cell contamination of chromosome 21 trisomy in a male fetus. For aneuploid samples with maternal contamination, the chromosomal ratio was between the values for normal samples and the values for trisomy samples depending on the degree of contamination. In the case of the contaminated sample chromosomal ratio for trisomy 21 was 1.193, while for euploid mean ratio was 0.981 and for regular trisomy 21 was 1.505.

## 3. Discussion

In this study, we developed a novel chip-based dPCR system for a rapid testing of the most common fetal aneuploidies. The method is based on the concept of digital PCR, which relies on the precise quantification of single template molecules without standards.

The digital PCR method has already been published for rapid analysis of the most common fetal aneuploidies. Fan et al. showed that the microfluidic dPCR is sufficiently precise to clearly detect fetal trisomy [17,18]. The study on 40 prenatal samples using microfluidic chambers-based approach in 6 h consisted of five reactions in which each copy number of target analyzed on chromosome 13, 18, 21, X and Y was referenced to copy number of target on reference chromosome 1.

In this concept study, for the first time we presented the use of chip-based QuantStudio 3D Digital PCR system as a new method for rapid fetal aneuploidies detection. The results obtained from 133 samples suggest that chip-based technology provides statistically significant discrimination between euploid and aneuploid samples. Moreover, the ROC curve analysis proved the potential utility of this system as a screening test. Our preliminary pilot study suggest that it was possible to obtain a significant difference between euploid and aneuploid samples according to the karyotype result. Thus, a larger cohort will be needed in order to determine an optimal threshold to classify samples and confirm the usefulness of the single-target analysis per chromosome.

The substantial pre-analytical risk for prenatal diagnostics is maternal cell contamination and fetal chromosomal mosaicism. Our result suggests that dPCR approach could detect aneuploidy mosaicism when the level of mosaicism was about 20%. Although, low grade mosaicism was not detectable with the current sensitivity, it was difficult to predict the clinical and phenotypic consequences of low mosaicism, especially placental mosaicisms. Thus, to determine the usefulness of our approach in mosaicism detection, further clinical research is necessary.

A chip-based approach workflow was straightforward, required limited pipetting steps and the results were obtained within 4 h. An additional advantage of this method is the risk minimization of contamination at the pre-PCR step, through tight closing of the reaction mixture on the chip (one-step procedure and not require post-PCR analysis). In comparison, the widely used droplet digital PCR system, requires additional multiple pipette transfers, which potentially increases the risk of contamination. In addition, droplet digital PCR is based on drop generation. That could be an extremely variable step negatively impacting the reliability of the results. In a chip-based dPCR system, the number of potential independent reactions is constant and equal to the number of wells (20,000). Data analysis is simple, does not require advanced calculations and therefore does not create additional interpretation problems. Furthermore, the equipment costs are two times lower than those of other available digital PCR platforms, so it is possible to implement QuantStudio 3D dPCR system with low setup cost in routine laboratory practice. Nevertheless, chip-based dPCR method has some limitations, for example, low throughput (only one sample per chip, although the potential capacity of thermocycler is up to 24 chips) and limited multiplexing, because only two types of probes per chip can be detected. However, in the setting of an average hospital facility for prenatal screening, the low throughput of this approach seems not to be a limitation.

Our results suggest that chip-based digital PCR was suitable for accurate and rapid determination of common fetal aneuploidies, providing minimum hands-on time methodology, reduced analysis time, cost-effectiveness and increased precision with absolute quantification. In our opinion the chip-based dPCR is a promising approach in routine laboratory testing.

## 4. Materials and Methods

### 4.1. Patient Enrollment and Sample Collection

For this study, 133 pregnant women at high risk of chromosomal abnormalities undergoing invasive prenatal diagnosis were recruited. For each pregnant woman, simultaneous analysis using QuantStudio 3D dPCR and a classical karyotyping was carried out. All patients signed an informed consent. All research and methods were performed in accordance with relevant regulations as approved by the Bioethics Committee of the Polish Mother’s Memorial Hospital Research Institute (74/2018, issue date: 11 September 2018). Used procedures followed the Declaration of Helsinki principles. The median (range) age of pregnant women undergoing invasive prenatal diagnosis was 34 (20 to 47) years. The median (range) age of gestation was 16 (11 to 33) weeks. Indications for invasive prenatal testing were: high maternal serum markers, ultrasound abnormalities, or other Indications. Details are provided in the Table 2.

### 4.2. DNA Extraction

Genomic DNA was extracted from amniotic fluid cells (4–6 mL) or chorionic villus samples. Amniotic fluid was centrifuged at 16,000× *g* for 5 min, supernatant was removed and cell pellet was dissolved in phosphate buffered saline (PBS). CVS were suspended in PBS. DNA was extracted and purified automatically using a MagCore (Cartridge Code: 110, running time: 38 min, elution volume: 60 μL) following the manufacturer’s instructions (RBC Bioscience, Taipei, Taiwan). Purified DNA was quantified using the NanoDrop spectrophotometer (Thermo Fisher Scientific, Foster, CA, USA) and stored at −20 °C until use.

### 4.3. Chip-Based Digital PCR Workflow

All 133 samples were processed by QuantStudio 3D Digital PCR system (Thermo Fisher Scientific, Foster City, CA, USA). DNA samples were detected and quantified using custom TaqMan^®^ assays labeled with the FAM or VIC fluorophore (Thermo Fisher Scientific). The probes in this study in highly conserved regions in the constant-copy genome were used as previously reported [16]. The target sequences did not coincide with common single-nucleotide polymorphisms or copy number variants. Primer sequences and probes are provided in Table A1. To avoid propagation of non-specific PCR products, fragments larger than 90 bp were amplified.

dPCR reactions were set up in a final volume of 15 μL, containing 7.5 μL of 2× QuantStudio 3D dPCR master mix, 0.75 μL of 20× FAM TaqMan^®^ assays, 0.75 μL of 20× VIC TaqMan^®^ assays and different DNA templates, each dissolved in nuclease-free water. To obtain the copy number of the target regions in the range 200–2000 copies/μL, the concentration of the input DNA was 10 ng/μL or less if the recommended concentration of DNA sample was not reached. The PCR samples were loaded onto chips with approximately 20,000 wells using QuantStudio 3D dPCR Chip Loader (Thermo Fisher Scientific). PCR amplification was performed in a ProFlex thermal cycler (Thermo Fisher Scientific) at the following standard thermal parameters: 96 °C for 10 min, 40 cycles of 60 °C for 2 min and 98 °C for 30 s, and then final extension at 60 °C for 2 min. For negative controls, water was added instead of genomic DNA. Positive controls for each assay were carried out by using euploid genomic DNA. Negative control chips were processed every day to exclude contamination. Following amplification, fluorescence was analyzed using the QuantStudio 3D dPCR Instrument. For each labeled sample, the detector read the fluorescence signals generated by FAM and VIC dye. Chip quality and data analysis were analyzed with QuantStudio 3D AnalysisSuite Cloud Software v3.1 (Thermo Fisher Scientific).

### 4.4. Statistical Analysis

Statistical analysis was performed using the Statistica 13.1 software (TIBCO). Chromosome ratios in euploid and aneuploid groups were compared using a Mann–Whitney *U* test. A ROC curve was then plotted and the area under it was calculated together with the corresponding 95% confidence interval. For slope ratios in mosaicism samples the Pearson’s correlation was calculated. Linear regression analyses were performed in order to obtain the slope values and correlation coefficients (*r*^2^). The threshold for declaring statistical significance was set at *p* < 0.05.

## 5. Patents

We had no patent applications or pending or awarded patents to declare. The method developed by us was free to be implemented and used in other laboratories.

## Figures and Tables

**Table 1:** Results obtained for duplex reactions using FAM and VIC-labeled probe assays.

Chr	Ratio of Slopes in Euploid Samples			Ratio of Slopes in Aneuploid Samples		
	Mean Ratio	Minimum Ratio	Maximum Ratio	Mean Ratio	Minimum Ratio	Maximum Ratio
13	1.012	0.935	1.145	1.507	1.392	1.508
18	0.950	0.915	0.980	1.424	1.382	1.463
21	0.981	0.955	1.008	1.505	1.429	1.533

**Table 2:** Clinical data and indications for invasive prenatal testing.

Indications	No. of Amniotic Fluid Samples (*N* = 106) ^2^	No. of Chorionic Villus Samples (*N* = 27) ^2^
Maternal age ^1^ (range)	34 ± 5.4 (20–47)	
≤35 years	57	10
>35 years	49	17
Gestational age ^1^ (range)	16 ± 3.8 (11–33)	
11–14 weeks	10	25
15–20 weeks	86	2
>20 weeks	10	0
Clinical characteristics		
Abnormal results of the first trimester screening ^2^	62	8
Abnormal results of ultrasound ^2^	45	20
Other indications	6	2

^1^ The maternal age and gestational age were presented as mean value ± standard deviation. ^2^ Several patients had more than one indication for prenatal testing.

## Abbreviations

NT	fetal nuchal translucency
β-hCG	free beta-human chorionic gonadotropin
PAPP-A	pregnancy-associated plasma protein-A
FISH	linear dichroism
QF-PCR	quantitative fluorescence PCR
MLPA	multiplex ligation-dependent probe amplification
dPCR	digital PCR
CVS	chorionic villus samples
ROC	receiver operating characteristic
PBS	phosphate buffered saline

## Appendix A

**Table A1:** Primer-probe assays. F, forward; R, reverse, VIC reporter dye-labeled probe with MGB quencher (minor groove binder) and FAM reporter dye-labeled probe with MGB quencher.

Gene	Chromosome Location	Primer and Probe Sequences (5′–3′)	Product Size (bp)
*MBNL2*	13q32.1	F: CTCACCTATCCACAATGCAA	81
		R: GGGATTCAAGCGAATTAACA	
		Probe: AGGTGCATCATGGGAACGGC-VIC	
*EHZF*	18q11.2	F: CCAGCTGGTACTTGGAAGAG	87
		R: TGTCGTATGTGGAGCCAAC	
		Probe: TCAGTGCCTGCCTGGTTCCC-FAM	
*PRDM15*	21q22.3	F: ATGTTTCGCCAACTTCTGAG	89
		R: AGAGCTATGGCACAAACCTG	
		Probe: TCCCAAACTCTCCTGCCCTGA-FAM	
*Noncoding*	Xp22.3	F: GATGAGGAAGGCAATGATCC	86
		R: TTGGCTTTTACCAAATAGGG	
		Probe: TGTTTCTCTCTGCCTGCACTGG-VIC	
*SRY*	Yp11.3	F: CGCTTAACATAGCAGAAGCA	84
		R: AGTTTCGAACTCTGGCACCT	
		Probe: TGTCGCACTCTCCTTGTTTTTGACA-FAM	
